# Supplementary material for: Natural Language Processing of Clinical Notes on Chronic Diseases: Systematic Review
Source: JMIR Med Inform. 2019 Apr 27;7(2):e12239. doi: 10.2196/12239 (PMC6528438; doi:10.2196/12239)
Supplement: Multimedia Appendix 1 [file medinform_v7i2e12239_app1.pdf]

Appendix 1. Search strategy

Scopus

1 ALL ("clinical notes" OR "medical notes" OR "clinical narratives")
2 ALL ("natural language processing" OR "medical language processing" OR "text mining" OR "information extraction")
3 ALL ("chronic disease" OR "heart disease" OR "stroke" OR "cancer" OR "diabetes" OR "lung disease"))
4 1 and 2 and 3
5 PUBYEAR AFT 2006 AND LANGUAGE (English)
6 DOCTYPE (ar)
7 4 and 5 and 6

Web of science

TOPIC:("clinical notes" OR "medical notes" OR "clinical narratives") AND TOPIC:("natural language processing" OR "medical language processing" OR "text mining" OR "information extraction") AND TOPIC: ("chronic disease" OR "heart disease" OR "stroke" OR "cancer" OR "diabetes" OR "lung disease")
Timespan: 2007-2018.

PubMed Keywords

((("clinical notes"[All Fields] OR "medical notes"[All Fields] OR "clinical narratives"[All Fields]) AND ("natural language processing"[All Fields] OR "medical language processing"[All Fields] OR "text mining"[All Fields] OR "information extraction"[All Fields])) AND ("chronic disease"[All Fields] OR "heart disease"[All Fields] OR "stroke"[All Fields] OR "cancer"[All Fields] OR "diabetes"[All Fields] OR "lung disease"[All Fields])) AND ("2007"[PDAT] : "2018/02/06"[PDAT])

ACM Digital Library

("Clinical notes" "clinical narratives" "medical notes")
+ ("natural language processing" "text mining" "medical language processing" "information extraction")
+("chronic disease" "heart disease" "stroke" "cancer" "diabetes" "lung disease")
All limited from January 1, 2007 to February 6, 2018.
